# Social Emotional Health Survey-Secondary (SEHS-S): A Universal Screening Measure of Social-Emotional Strengths for Spanish-Speaking Adolescents

**DOI:** 10.3390/ijerph16244982

**Published:** 2019-12-07

**Authors:** Jose A. Piqueras, Tiscar Rodriguez-Jimenez, Juan Carlos Marzo, Maria Rivera-Riquelme, Agustin E. Martinez-Gonzalez, Raquel Falco, Michael J. Furlong

**Affiliations:** 1 Department of Health Psychology, Faculty of Social and Health Sciences, Campus of Elche, Miguel Hernandez University (UMH), 03202 Elche, Spain; jc.marzo@umh.es (J.C.M.); maria.rivera.riquelme@gmail.com (M.R.-R.); rfalco@umh.es (R.F.); 2 Department of Psychology, Campus of Los Jerónimos, Catholic University of Murcia (UCAM), 30107 Murcia, Spain; trodriguez@ucam.edu; 3 Department of Developmental Psychology and Didactics, Faculty of Education, Campus of San Vicente del Raspeig, University of Alicante (UA), 03690 Alicante, Spain; agustin.emartinez@gcloud.ua.es; 4 International Center for School Based Youth Development, Department of Counseling, Clinical, and School Psychology, Gevirtz School, University of California, Santa Barbara (UCSB), CA 93106-9490, USA; mfurlong@ucsb.edu

**Keywords:** adolescents, positive mental health, social and emotional health survey-secondary, Covitality, measurement

## Abstract

The Social Emotional Health Survey-Secondary (SEHS-S), which is a measure of core psychological assets based on a higher-order model of Covitality, is comprised of 36 items and four latent traits (with three measured subscales): belief in self (self-efficacy, self-awareness, and persistence), belief in others (school support, family coherence, and peer support), emotional competence (emotional regulation, behavioral self-control, and empathy), and engaged living (gratitude, zest, and optimism). Previous international studies have supported the psychometric properties of the SEHS-S. The present study extended this research by examining the psychometric properties of a Spanish-language adaptation with a sample of 1042 Spanish adolescents (*M*_age_ = 14.49, *SD* = 1.65.). Confirmatory factor analyses replicated the original factorial structure, with hierarchical omega between 0.66–0.93, with 0.94 for the total score. Factorial invariance across genders revealed small latent mean differences. A path model evaluated concurrent validity, which revealed a significant association between Covitality and bidimensional mental health (psychological distress and well-being). Specifically, correlational analyses showed a negative association with internalizing/externalizing symptoms, and positive associations with subjective well-being, health-related quality of life, and prosocial behaviors. This study provides an example of a culturally relevant adaptation of an international tool to measure student strengths, which is critical to planning school programming and policy.

## 1. Introduction

The World Health Organization (WHO) states that mental health is not merely the absence of psychological symptoms, but mental health encompasses holistic mental and social well-being [1]. This contrasts with traditional clinical research that has focused on diagnosing the presence or absence of mental disorders [2], and which has given relatively less attention to indicators of positive mental health [3,4,5]. Consequently, while WHO recognizes the need to prevent mental or psychological distress, it recommends concurrent efforts to promote psychological well-being as part of a complete mental health orientation [1].

An approach to operationalizing youths’ complete mental health is provided by the Bidimensional Mental Health Model (BMHM), first suggested by Greenspoon and Saklofske [6]. The BMHM proposes a measurement and classification approach that involves both psychological distress and subjective well-being. Thus, in the context of clinical and research practice, and contrary to the traditional approach (one-dimensional and markedly biomedical), a balance is sought between assessment and intervention on risk factors and psychopathology, as well as protective factors and psychological well-being. Subjective well-being and psychological distress are considered two opposite and independent phenomena, in such a way that an increase in distress does not imply a directly proportional decrease in well-being. This model has shown incremental validity in predicting concurrent outcomes compared with models that include only distress or psychopathology indicators [7,8,9,10]. It is further recognized that educational institutions are a primary setting in which mental health promotion programs based on the BMHM are developed and implemented [11,12]. Many different authors have indicated that school-based screening measures can play a critical role for the dual purpose of fostering all students enhanced complete mental wellness while simultaneously providing early identification of children who need mental health services before problems arise and become more difficult to address [13,14].

In the BMHM context, interest has grown for validated comprehensive strength-based assessment models for school settings. One of those models is the Furlong, You, Renshaw, Smith, and O’Malley’s Covitality integrated social emotional mindset model [15]. This model has a growing interest in positive educational psychology [16] and the co-occurrence of students’ positive strengths and how these strengths in combination contribute to global well-being. The Covitality model suggests that the combination of positive psychological characteristics and their synergic effects is more important than any individual positive characteristic for positive youth development and better well-being [15].

### 1.1. Social Emotional Health Survey–Secondary (SEHS-S)

The Social Emotional Health Survey System (SEHS-Sys) [15] was developed to measure the components of the Covitality latent construct among youth. The SEHS is a universal screening instrument for assessing students’ Covitality, which allows for the examination of the relations of Covitality with school outcomes. The SEHS has three versions: primary, secondary, and higher education. In the present study, we focus on the Social Emotional Health Survey–Secondary (SEHS–S) [15], which is appropriate for using with adolescents ages 12 to 18 years old. The SEHS-S includes 36 items for the assessment of core psychosocial assets based on a higher-order model comprised of 12 first-order and four second-order latent traits, and a higher-order general factor (called Covitality). The first domain, called belief-in-self, consists of three subscales: self-efficacy, self-awareness, and persistence. The second domain, called belief-in-others, is comprised of three subscales: school support, peer support, and family support. The third domain, known as emotional competence, consists of three subscales: emotion regulation, empathy, and behavioral self-control. Engaged living, which is the final domain, is comprised of three subscales: gratitude, zest, and optimism. For 10 of the 12 subscales, the students’ responses are recorded using a four-point scale (1 = not at all true of me, 2 = a little true of me, 3 = pretty much true of me, and 4 = very much true of me). The subscales for measuring gratitude and zest used a five-point response scale: (1 = not at all, 2 = very little, 3 = somewhat, 4 = quite a lot, 5 = extremely) [15] (a brief description of SEHS-S subscales is available as Online Supplemental Material for this article).

### 1.2. Covitality Psychometric Support

An increasing number of studies have provided information about the psychometric properties of the SEHS-S, including for the validity of the higher-order model, invariance across sociocultural and gender groups, reliability (internal consistency), and validity evidence (construct, predictive, and convergent, among others). Previous studies reported evidence supporting the reliability and validity of the higher-order measurement model by means of Confirmatory Factor Analyses [15,17,18,19]. Each study replicated the same higher-order structure with high factor loadings (all in the 0.50–0.91 range). Following from previous CFAs, evidence has supported measurement invariance for gender [15,17,18], younger and older adolescents [19], and five ethnic groups (Latino, White, Asian, Black, and multiethnic) of California students [20].

Reported internal consistency reliabilities have been favorable across previous studies: belief in self (0.75–0.84), belief in others (0.81–0.87), emotional competence (0.78–0.82), engaged living (0.87–0.88), and Covitality (total score across the 36 items, 0.91–0.95). Concerning different cultures and countries, for instance, internal consistency estimates (Cronbach’s alpha) for the total SEHS-S Covitality score were 0.93 for a sample of Japanese youth and 0.94 for a sample of South Korean youth, which was comparable to a U.S. sample (0.95) from one of the initial validation studies [20].

Additionally, previous studies have examined the associations among Covitality and adolescents’ bidimensional mental health. For adolescents, these studies evaluated structural models that examined the associations among the four observed second-order SEHS-S factors, the hypothesized first-order Covitality construct, and a composite mental health index with all models showing good fit to the data [15,17,18].

Some previous studies have reported intercorrelations between SEHS-S subscales [21,22,23,24,25,26,27]. The lowest intercorrelations between the four second-order factors were found for the association between emotional competence and engaged living (0.25–0.63) and the highest one for belief-in-self and engaged living (0.44–0.72). Regarding the correlations between the 12 first-order subscales, studies indicated values between 0.11 and 0.57 [23] and between 0.11 and 0.61 in Turkey and between 0.16 and 0.67 in USA [27]. This last study also found correlations between 0.54 and 0.68 in Turkish teenagers and between 0.53 and 0.74 in North Americans. The analysis of these correlations is important since they did not exceed 0.70 in any case, which could be interpreted as the absence of multicollinearity. Additionally, the correlations between the four factors and the Covitality total score were between 0.62 and 0.83, with the highest one found for engaged living (0.76–0.83) and the lowest one for emotional competence with Covitality (0.62–0.66).

Other studies have examined SEHS-S convergent validity with other indicators of youth global well-being, life satisfaction, quality of life, school adjustment, and prosocial behavior (*r* = 0.36 to 0.89) [15,17,18,27], and negatively correlated with measures or youth distress, such as diverse measures of behavioral and emotional symptoms (*r* = −0.08 and −0.63) [15,17,18,19,24,27,28]. The SEHS-S was significantly and positively correlated with the Strengths and Difficulties Questionnaire (SDQ) [29] prosocial behavior subscale (*r* = 0.40) [27], with subjective well-being among Korean youths (*r* = 0.56) [18] and among Californian teens (*r* = 0.57) [30], and Turkish adolescents (*r* = 0.66) [27]. Additionally, other analyses have indicated that the SEHS-S was negatively correlated with depression, anxiety, and stress (*r* = −0.22 to −0.36) in Chinese youths [28], with behavioral and emotional symptoms in USA and Latin American adolescents (*r* = −0.63) [20], and with the SDQ total difficulties scale (*r* = −0.25) among Turkish [27] and Korean youths (*r* = −0.08 to −0.25) [24]. Lastly, analyses have shown that the SEHS-S has significant positive relations with school outcomes, such as school adjustment, prosocial behavior, academic performance, school safety, and lower risk of identifying as a gang member [15,24,31]. The previously mentioned studies have examined the psychometric properties of the SEHS-S and the measurability of the core components of Covitality in secondary school samples from many different countries and cultures such as China [28], Korea [18], United States [15,19,23], Japan [17], Turkey [27], and five California sociocultural groups (Latino, Black, blended heritage, Asian, and White) [20].

In spite of all the evidence accumulated in the use of the SEHS, no study has examined the viability of the SEHS-S to assess positive psychological mindsets within Hispanic cultural contexts other than those from the U.S. Given that the use of the same language does not guarantee the equivalence of all linguistic expressions and their meanings, and cultural differences may exist, there is a need to examine the viability of the SEHS-S for different Spanish-speaking contexts. Furthermore, Chen [32] observed that when a survey is used with a cultural group for which it was not originally intended, measurement invariance should be rigorously tested to ensure that any conclusions drawn about group differences are not simply artifacts of measurement error. Additionally, the Spanish language is the second most spoken language in the world, which provides a compelling rationale to examine the psychometric properties of the SEHS-S for use within Hispanic cultural contexts.

### 1.3. Study Aims and Contributions

Consequently, the aim of this study was to investigate the psychometric properties of a Spanish-language adaptation of the SEHS-S, which provided additional evidence about the cross-cultural utility of the SEHS-S Covitality model and, thereby, contributed to future cross-cultural research of adolescents’ social-emotional health. In making this contribution to the literature, we provide the following analyses of the SEHS-S Spanish version: factor structure, invariance for gender groups, reliability, and evidence of validity such as the relations of SEHS-S subscales with indicators of mental health.

## 2. Materials and Methods

### 2.1. Participants

Participants were recruited from eight secondary education institutes (three subsidized and five public schools) located in the Alicante province in Spain. A convenience sample of 1060 students was generated from schools’ that voluntary agreement to participate in the study and that had access to computer resources. A few cases were not included in the analyses when they did not indicate gender (*n* = 9) or were 11 (*n* = 6) or more than 18 years old (*n* = 3). The large sample (S1) used in this study’s analyses had 600 (57.6%) males and 442 (42.4%) females, aged between 12 to 18 years (*M* = 14.49, *SD* = 1.65). These students belonged to a compulsory secondary education level, comprising from Grade 7 to 10 (first to the fourth year of secondary education in Spain), and at High School, including Grades 11–12 (first and second year of high school in Spain). Secondary education from 12 to 16 years old is compulsory in Spain (seventh to tenth year) and high school is two not compulsory years of preparation for university studies (Bachillerato in Spain). For the validity analyses, we used a subsample (S2) of 222 students (*M_age_* = 15.43, *SD* = 1.62, age range: 12–18, 53.6% males).

### 2.2. Variables and Instruments

#### 2.2.1. Covitality

Social and Emotional Health Survey (SEHS-S). The 36 SEHS items are available as Online Supplemental Material for this article. The description and the summary of psychometric properties of SEHS-S was included in the introduction section. The internal consistency coefficients for S1 and S2 are presented in the Results section.

The Spanish version of SEHS-S was developed in accordance with the guidelines of the International Test Commission [33], using an iterative-translation method that began with several independent translations. The item translations were then reviewed by a joint committee comprised of translators with knowledge of Spanish language and culture and specialists in the field of assessment who analyzed the adequacy of the adapted version. Any translation discrepancies arising were discussed and appropriate corrections were made to the item translations. Consensus on translated item wording in Spanish was achieved.

#### 2.2.2. Brief Mental Health Measures

Strengths and Difficulties Questionnaire (SDQ) [29]. The SDQ assesses different emotional and behavioral problems in children and adolescents from 11 to 17 years old. It is a 25-item questionnaire distributed across five subscales: emotional symptoms, conduct problems, hyperactivity, peer problems, and prosocial behavior. Emotional and peer subscales can be combined into an internalizing subscale and the behavioral and hyperactivity subscales into an externalizing subscale (alongside the fifth prosocial subscale) [34]. We used the Spanish version taken from the Strengths and Difficulties Questionnaire (SDQ) website (see, http://www.sdqinfo.com/). The SDQ uses a three-option response format (0 = not true, 1 = somewhat true, 2 = certainly true). The internal consistency for this study’s sample was similar to those reported in previous studies [11,35,36]. In S2, Omega hierarchical was 0.73 for externalizing symptoms, 0.80 for internalizing symptoms, 0.78 for prosocial behavior, and 0.80 for the total score.

#### 2.2.3. Subjective Well-Being

Mental Health Continuum-Short Form (MHC-S) [37]. The MHC-SF consists of 14 items, providing the frequency (in the past month) that students experienced levels of positive mental health or well-being. Three items (happy, interested in life, and satisfied) represent emotional/hedonic well-being, six items represent psychological well-being, and five items represent social well-being. These 11 items represent eudemonic well-being. This test has received psychometric support for its use with adolescents with subscale and total score Cronbach alpha coefficients all above 0.80, which indicates good reliability [38,39]. In S2, the reliability was 0.89 for general well-being, 0.72 for emotional/hedonic well-being, and 0.87 for eudemonic well-being (0.77 psychological and 0.81 social well-being).

#### 2.2.4. Health-Related Quality of Life

Kidscreen-10 Index or KIDSCREEN-10 [40]. This is a 10-item questionnaire that assesses subjective Health-Related Quality of Life (HRQL) and well-being for children and adolescents ages 8 to 18 years during the previous week. For each item, five response options are provided: “not at all”, “slightly”, “moderately”, “very”, and “extremely”. The index addresses affective symptoms of depressed mood (e.g., Have you felt sad?), cognitive symptoms of disturbed concentration (e.g., Have you been able to pay attention?), psycho-vegetative aspects of energy and feeling well (e.g., Have you felt full of energy?), and psychosocial aspects related with mental health, such as the ability to experience fun with friends or getting along well at school (e.g., Have got on well in school?). The reliability of the KIDSCREEN-10 in S2 was 0.80, which is equivalent to that reported by Erhart et al. (α = 0.81) [41].

### 2.3. Procedure

Since 2011–2012, the Covitality-Spain team has been implementing psychological assessment practices including strengths and difficulties in adolescents. For the present study, parents and students were informed that participation was voluntary and anonymous. All participants gave their informed consent for inclusion before they participated in the study. The study was conducted in accordance with the Declaration of Helsinki, and the protocol was approved by the Ethics Committee of the university of the first author (Project identification code: DPS.JPR.02.17).

The survey instrument was administered online to students schoolwide within a universal prevention framework [11]. In the present study, we used the web-based assessment protocol for BMHM of children and adolescents, the DetectaWeb Project (for further details, see its description in Piqueras et al.) [11]. The convenience sample of recruited students were organized within their respective centers in order to complete the questionnaire in their schools’ computer rooms. The researcher responsible for the groups were previously trained in the application of the assessments and received online guidance. All the participants completed the SEHS-S. In order to carry out validity analyses, we used the subsample S2 that also completed complementary instruments. The S2 completed 85 items (SEHS-S, SDQ, MHC-SF, and Kidscreen-10). The average time taken to complete the S2 survey was 30 min.

### 2.4. Data Analyses

The statistical analyses were carried out using the following applications SPSS 24 (IBM corp., Armonk, NY, USA) for Windows and EQS 6.3 (Multivariate Software, Inc., Temple City, CA, USA). An initial exploratory analysis was performed to examine the presence of atypical cases and to evaluate the univariate and multivariate normality assumption. To obtain evidence of the instrument’s construct validity in a Spanish sample (S1), we tested the original higher-order factor model proposed by Furlong et al. [15] as well as other alternative models, according to DiStefano, Greer, and Kamphaus [42], such as unidimensional, correlated, and bifactor model, using the confirmatory factor analysis (CFA) procedure. The analyses were carried out using the method of robust maximum likelihood (Robust ML). We reported the following indices: chi-square (χ^2^), Satorra Bentler Chi-square (S-B χ^2^), robust Root Mean Square Error Approximation (RMSEA), Comparative Fit Index (CFI), Non-Normed Fit Index (NNFI), Goodness of Fit Index (GFI), and Standardized Root Mean Square Residual (SRMR). For RMSEA, values less than 0.05 indicate a good fit model [43]. The CFI, NNFI, and GFI values indicate good fit with values greater or equal to 0.95 and acceptable fit when they are higher or equal to 0.90 [44], while the SRMR values are good with lower values to 0.08, and it is considered acceptable when values approach 0.001.

In order to compare the latent means differences by gender, a model with Covitality as a second-order factor was considered. Factorial invariance of the model (FI) was analyzed following the procedure suggested by Byrne [45], according to which the measurement invariance applies to (a) validity of the configural model (M1, base line model), (b) metric invariance (equal factor loadings across groups, M2: M1+ first-order factor loading, M3: M2+ Second order factor loading), and (c) scalar invariance (equal item intercepts across groups, M4: M3+ observed variable intercepts, and M5: M4+ latent factors intercepts). When the strong measurement invariance (metric and scalar) is reached, the comparison of latent means is justified. According to the methodology proposed by Cheung and Rensvold [46], we reported CFI, ∆CFI, Gamma hat, ∆Gamma Hat, McDonald’s Non-Centrality Index (NCI), and ∆McDonald’s NCI. A value of ∆CFI smaller than or equal to −0.01 indicates that the null hypothesis of invariance should not be rejected. For the Increment of Gamma Hat and the Increment of McDonald’s NCI, the critical values are −0.001 and −0.020, respectively. After these considerations, the calculations to compare the latent means across gender were carried out.

The values of internal consistency were calculated with RStudio [47] software, using the Psych [48] and GPArotation [49] packets. We calculated Omega hierarchical and Coefficient H as alternatives to Cronbach’s alpha, which is a measure of internal consistency reliability questioned recently in the statistical literature because it is not the optimal method for reporting on reliability [50].

The association between variables was also calculated, and the magnitudes of the associations were interpreted, according to the criteria proposed by Cohen [51]. According to Cohen’s power primer [52], *r* less than or equal to 0.10 should be considered small, a *r* around 0.30 belongs to a medium, and *r* equal to or greater than 0.50 would be considered as a large effect size.

## 3. Results

### 3.1. Confirmatory Factor Analysis

Using the S1 of students, confirmatory factor analysis was conducted to examine the higher-order structural model that was described in previous SEHS-S validity studies, i.e., Reference [15] as well as other alternative models, according to DiStefano et al. [42]. Model fit indices for the SEHS-S are presented in Table 1.

**Figure 1:**
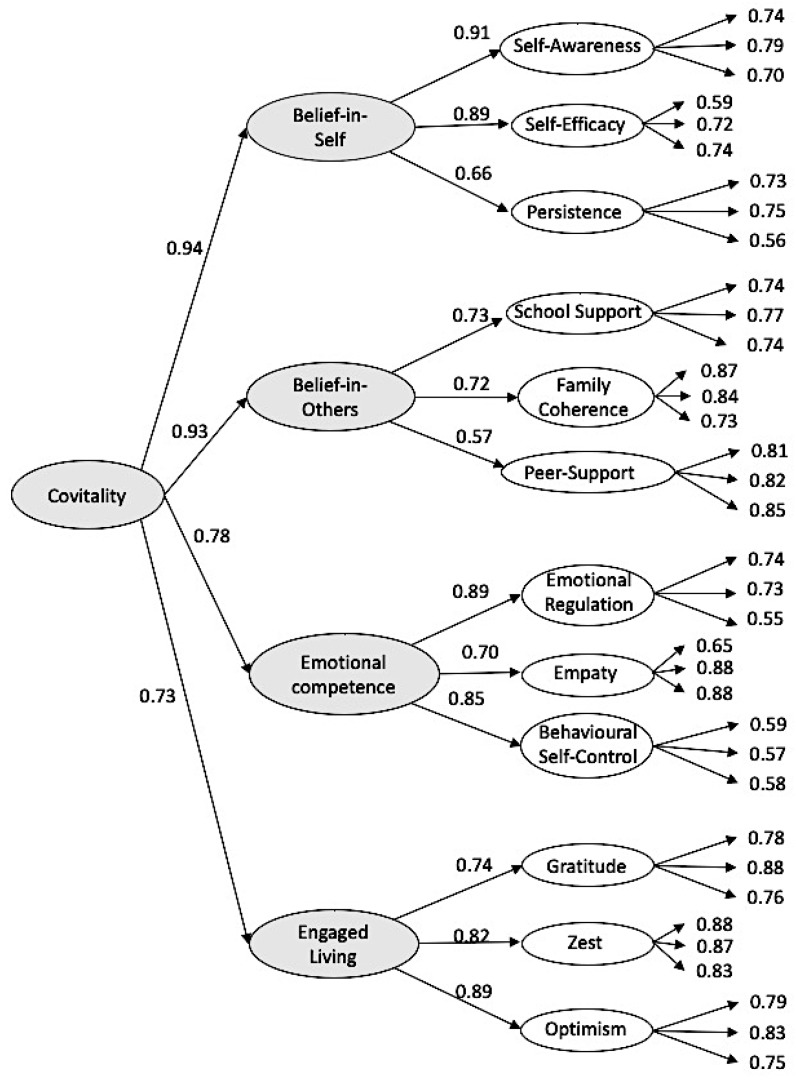
Standardized factor loadings for the Social Emotional Health Survey-Secondary (Sample 1).

With this result, it can be concluded that the unidimensional and the bifactor models inadequately fits the data. Both the correlated model and the third-order factorial structure showed adequate fit values. Given that there were few differences between them, and considering that the higher-order model of the SEHS-S makes it possible to determine the role of Covitality (as a higher-order factor) in determining its different factors, as well as the fact that this model is in line with previous works on Covitality, we chose the third-order model because, from the theoretical point of view, it is a more appropriate model. This higher-order structural model indicates that Covitality influence over the four SEHS domains (belief in self, belief in others, emotional competence, and engaged living). The domains load over the 12 subscales, which were composed by three items each show adequate fit values: S-Bχ^2^ = 1065.86, *df* = 573, *p* < 0.01, CFI = 0.96, NNFI = 0.957, GFI = 0.923, SRMR = 0.050, RMSEA = 0.029, and 90% CI [0.026,0.031]. Due to the result that the RMSEA is below 0.05, CFI and NNFI are greater than 0.95. GFI is greater than 0.90 and SRMR is exactly 0.05. It can be concluded that the model fits adequately. The standardized parameter values of the survey items varied between 0.56 and 0.79 in the dimension of belief in self, 0.73 and 0.87 in belief in others, 0.55 and 0.88 in emotional competence, and 0.75 and 0.88 in the dimension of engaged living (see Figure 1).

### 3.2. Factorial Invariance (FI)

Since Mardia’s test presented violation of the multivariate normality, a robust estimation method was used to evaluate FI by gender: Maximum Likelihood (Robust ML). Subsequently, the fit of the model for both samples was calculated. The model adequately fit the data for males: χ^2^ = 1212.30, S-Bχ^2^ = 850.68, *df* = 573, *p* < 0.01, CFI = 0.962; SRMR = 0.052; RMSEA = 0.028, 90% CI [0.024, 0.032] and for females: χ^2^ = 1050.65, *df* = 573, *p* < 0.01, S-Bχ^2^ = 834.75; *df* = 573, CFI = 0.950, SRMR = 0.058, RMSEA = 0.032, and 90% CI [0.027, 0.037].

The next step was to test the metric invariance or equivalence of factorial loadings between the two groups (weak invariance). Hence, the unrestricted multi-group model (M1) was first calculated. The model was calculated by establishing equality of factorial loadings of the two samples (M2). Following Cheung and Rensvold [46] recommendations, a value of ∆CFI smaller than or equal to −0.01, a value of ∆McDonald’s NCI smaller than or equal to −0.001, and a value of ∆GHI lower than or equal to −0.020 indicate that the null hypothesis of invariance should not be rejected. Table 2 shows that all differences for CFI, McDonald’s NCI, and GHI between model 1 and the rest of the models are smaller than critical values, or the difference is positive. The means that fit for the second model in the comparison are better. Hence, the results provide evidence that the second-order Covitality model had sufficient invariance across genders.

### 3.3. Gender Differences

The fit of the model for comparing gender differences was good: χ^2^ = 601.44, S-Bχ^2^ = 474,58, *df* = 118, *p* < 0.01, CFI = 0.930; SRMR = 0.078, RMSEA = 0.063, 90% CI [0.055, 0.071]. Given that measurement invariance was established, we tested for latent mean differences. To accomplish this, mean differences were examined across genders, with males set as the reference group. Results showed significant latent mean group differences in all factors, except for belief-in-others (Factor intercept = 0.068, SE = 0.039, *p* > 0.05, *d* = 0.11). Belief-in-self (Factor intercept = −0.131, SE = 0.038, *p* < 0.01, *d* = 0.21) and engaged living (Factor intercept = −0.124, SE = 0.050, *p* < 0.01, *d* = 0.15) had higher means for males, and emotional competence for females (Factor intercept = 0.077, SE = 0.035, *p* < 0.01, *d* = 0.13). Covitality was higher in males (Factor intercept = −0.088, SE = 0.017, *p* < 0.01, *d* = 0.34). As can be seen, the effect sizes (*d*) found were small for first-order factors, and near-medium for the second-order factor (Covitality).

### 3.4. Reliability

The omega hierarchical coefficient was calculated to examine the reliability of the SEHS-S. For the S1, internal consistency was 0.94 for the total score of Covitality, 0.88 for belief-in-self (self-efficacy = 0.79, self-awareness = 0.85, and persistence = 0.77), 0.89 for belief-in-others (school support = 0.86, family coherence = 0.91, and peer support = 0.93), 0.86 for emotional competence (emotional regulation = 0.78, empathy = 0.89, and behavioral self-control = 0.66), and 0.92 for engaged living (gratitude = 0.87, zest = 0.92, and optimism = 0.91).

For S2, internal consistency was 0.87 for the total score of Covitality, 0.82 for belief-in-self (self-efficacy = 0.71, self-awareness = 0.80, and persistence = 0.74), 0.77 for belief-in-others (school support = 0.76, family coherence = 0.90, and peer support = 0.91), 0.80 for emotional competence (emotional regulation = 0.65, empathy = 0.86, and behavioral self-control = 0.61), and 0.89 for engaged living (gratitude = 0.75, zest = 0.86, and optimism = 0.88).

### 3.5. Path Model for Bidimensional Mental Health

**Figure 2:**
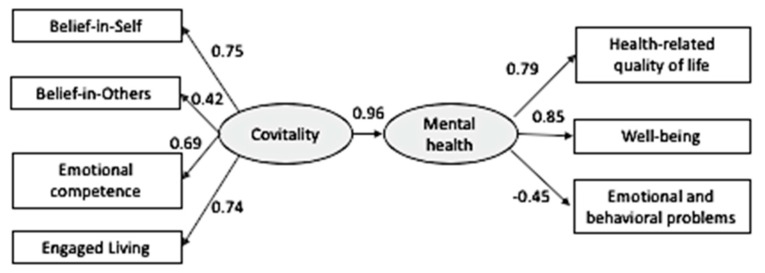
Bidimensional mental health and Covitality model underlying the Social and Emotional Health Survey (Sample 2).

**Figure 3:**
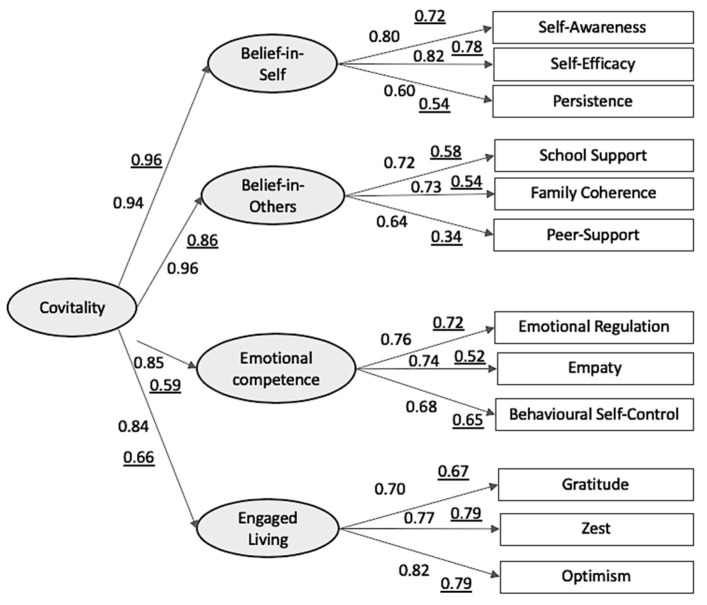
Covitality model underlying the Social and Emotional Health Survey (Sample 1).

To further examine the associations among the four observed second-order SEHS-S factors, the hypothesized first-order Covitality construct, and adolescents’ bidimensional mental health outcomes, a structural model was conducted from Covitality to the outcome variable (the BMHM-based composite score; see Figure 2). As expected, the analysis revealed a significant positive relation to mental health outcomes with the overall model having good fit to the data, S-Bχ^2^ = 36.18, *df* = 13, *p* < 0.001, SRMR = 0.043., RMSEA = 0.090, 90% CI [0.056, 0.125], CFI = 0.95, and GFI = 0.95. Figure 3 presents the standardized coefficients of the final path model.

### 3.6. Relations of SEHS-S Subscales with Indicators of Mental Health

We analyzed intercorrelations among the SEHS-S subscales (see Table 3) to evaluate evidence of convergent and discriminant validity. Overall, the correlations among all subscales scores (12 of first-order, 4 of second-order, and 1 of third-order factors) were significant, with only three exceptions: (a) peer support-persistence, (b) behavioral self-control-family coherence, (c) and optimism-persistence). Among the 12 first-order factor scores, the intercorrelations were between 0.11 and 0.56. The associations between engaged living subscale scores were the highest (0.52–0.56) and were lowest for the belief in others subscales (0.14–0.16). Concerning the four second-order factors, the inter-correlations were between 0.35 and 0.56. Lastly, all first-order (*r* = 0.46–0.71) and second-order (*r* = 0.65–0.83) scales had a strong association with SEHS-S Total Covitality.

Regarding evidence of convergent and discriminant validity, analyses with S2 provided positive correlations between Covitality and measures of positive variables (well-being, health-related quality of life, and prosocial behaviors), as well as negative associations with internalizing and externalizing symptoms. The magnitudes of the associations were large for well-being outcomes and medium for distress indicators (see Table 4).

## 4. Discussion

The aim of this study was to analyze the psychometric properties of SEHS-S with Spanish-speaking adolescents from Spain. As described in this section of the article, the results of this study supported the validity of the Spanish version of SEHS-S as a tool for evaluating the components of Covitality with adolescents.

### 4.1. Structural Validity

The results of the confirmatory factor analysis of the Spanish version of the SEHS-S in the adolescent sample supported the factor structure reported for the original U.S. version [15], for five different sociocultural groups in California [20], for a Korean sample [18], for a Japanese sample [17], and for a Turkish sample [27]. In addition, the invariance analyses suggested that the Spanish SEHS-S measures the same latent traits in the same way in males and females, as reported in previous studies [15,17,18,20,27]. The comparison of latent means for males and females in the present study indicated that differences were significant with small-medium to near-medium effect sizes in favor of female adolescents. These data are consistent with previous studies indicating that when gender differences are found, females are more likely to have higher scores on belief-in-other and emotional competence, while males are more likely to strongly endorse belief-in-self and engaged living with small to medium effect sizes [15,17,18,20].

### 4.2. Reliability

The reliability estimates showed that the alpha coefficients for the four first-order factors were all above 0.82. Hence, the reliability is favorable and these findings were similar to the values found in previous studies [17,18,19,27]. The alpha coefficient of 0.93 for the Covitality index is comparable to that reported in previous studies, which were in the range of 0.89–0.95 [15,17,18,19,20,27].

### 4.3. Relations of SEHS-S Subscales with Indicators of Mental Health

The tested path model showed a significant positive relation between the four observed second-order SEHS-S factors, the hypothesized third-order Covitality construct, and adolescents’ BMHM composite score. This finding is consistent with previous results reported by Furlong et al. [15], Ito et al. [17], and Lee et al. [18], which showed good fit to path models including SEHS-S and indicators of well-being/mental health.

We then examined the intercorrelations among the SEHS-S subscale scores. The correlations between the 12 first-order latent traits were from 0.11 to 0.56, with only three non-significant exceptions (peer support-persistence, behavioral self-control-family coherence, and optimism-persistence). These results are consistent with previous studies reporting correlations ranging from 0.11 to 0.57 [23], from 0.11 to 0.61 in Turkey, and from 0.16 to 0.67 in USA [27]. This last study also found correlations between 0.54 and 0.68 in Turkish teenagers and between 0.53 and 0.74 in North Americans. 

Concerning the four second-order factors, our data showed correlations between 0.35 and 0.56. In fact, we found the similar lowest intercorrelations (*r* = 0.35) between emotional competence and engaged living as in previous studies (*r* = 0.25 to 0.63) and the similar highest one (*r* = 0.56) for belief in self and engaged living (*r* = 44 to 72) [21,22,23,24,25,26,27].

Lastly, the correlations between the four factors and the Covitality total score in our S2 were consistent (*r* = 0.65 to 0.83) with previous studies (*r* = 0.62 to 0.83), with the highest one found for Engaged living (our sample: *r* = 0.83; previous studies: *r* = 0.76 to 0.83) and the lowest one found for Emotional Competence with Covitality (our sample: *r* = 0.65, previous studies: *r* = 0.62 to 0.66) [21,22,23,24,25,26,27].

With respect to concurrent validity, the analyses showed positive correlations between Covitality and well-being measures, health-related quality of life, and prosocial behavior. Negative correlations were with general distress, and internalizing and externalizing symptoms. These results are consistent with previous literature reporting positive correlations (*r* = 0.36–0.89) between Covitality and subjective well-being, life satisfaction, quality of life, school adjustment, prosocial behavior, and positive cognitive and emotional regulation [15,17,18,27], as well as negative correlations (*r* = −0.22 and −0.63) with internalizing and externalizing symptoms, such as depressive symptoms and behavioral problems [15,17,18,27]. These findings support the use of the Covitality index as a general indicator for positive social-emotional development of youth.

## 5. Conclusions

Although the findings found are promising, limitations to this work should be considered in future research. Since the SEHS-S is a self-report scale, these data may be affected by social desirability and other response biases, since responses to positive construct questions may generate desirable responses. The opposite is true for more negative or stigmatizing questions, such as those regarding symptoms. To investigate this possibility, future studies should be directed to examine test-retest reliability. It is also necessary to use randomized and longitudinal samples to analyze the stability of Covitality during adolescence and to better understand how Covitality might function as a protective factor against the effects of stressful life events in adolescence. A final point was that only subsample S2 completed different measures in this study, depending on the conditions of time agreed with each center. Although we would have preferred both samples completing the same number of measures, we considered that it could have caused a response burden on the students due to the fact that the survey would have been too long and then fatigue and related factors could have produced lower data quality.

A main practical implication of this study for researchers and professionals is a general observation about the convenience of using BMHM-based screening/monitoring in Spanish secondary schools. Since the use of the BMHM approach has shown incremental validity in predicting concurrent outcomes compared with models including only distress or psychopathology [9,10,12]. The use of this type of balanced, comprehensive, and complete assessment offers an alternative assessment approach for screening and monitoring youth mental health.

The application of measures, such as SEHS-S, that consider distress or symptoms of psychopathology in combination with positive indicators of thriving social-emotional health is a step forward and is clearly applicable to both school and clinical contexts. In addition, performing evaluations that are not only focused on the presence of problems is an advance for any psychological evaluation that from a clinical, school, or health point of view can help young people. This is particularly salient in school contexts that are specifically dedicated to promoting well balanced youth development across physical, cognitive, psychological, and social domains. Consequently, the SEHS-Secondary has the potential to provide a valid, innovative, and useful instrument when employing the BMHM assessment model with adolescents. This instrument helps address the need for comprehensive assessment of complete mental health in schools. In addition, the SEHS-S can provide useful data for developing prevention programs for adolescents’ mental health, for improving early detection for high-risk children and adolescents at school, and for promoting the well-being and psychological skills that enhance mental health.

Specifically, the use of SEHS-S, according to the Covitality model approach, focuses not only on assessment and warning when problems are detected, but also on the examination of social emotional strengths both at the group level and individual level. Therefore, this approach can be useful because secondary schools can use these data to help students both individually and, at the same time, to take a snapshot of the whole student body in order to plan universal programs and services. For example, at the individual level, SEHS-S may suggest that a particular student may benefit from individual action to promote the development of his personal resources. Simultaneously, the data from the group assessment can provide information as relevant since boys belonging to the first grade of Spanish secondary education (equivalent to seventh grade in U.S.) in a specific school do not feel particularly connected to the school, which suggests that a specific intervention might allow for an increase in school belonging during the transition to a new school. Lastly, individual and group information can be used in a complementary way to help school psychologists and practitioners direct resources in ways that are appropriate for both students and school systems. In addition, regular, systematic administration of the SEHS-S provides information that could help increase awareness of the need for programs and services that aim to foster the development of social-emotional competencies and more broadly positive education practices. It could also provide data to evaluate the changes that occur in school systems as a consequence of specific actions or school policies.

In conclusion, this study supports the use of SEHS-S to better understand and promote positive student development, and could facilitate cross-cultural studies by making the Spanish version of the questionnaire available. This scale could be useful for assessing the socio-emotional health of students and clarifying their individual needs in order to promote school programs and foster these core youth competences.

## Figures and Tables

**Table 1:** Model Fit Indices for the SEHS-S. Sample 1.

	S-Bχ^2^	RMSEA (90% CI)	NNFI	CFI	SRMR	GFI
Model A	6627.17 (594)	0.990 (0.097, 101)	0.489	0.518	0.093	0.612
Model B	920.83 (572)	0.024 (0.021, 027)	0.969	0.972	0.048	0.924
Model C	1065.86 (573)	0.029 (0.026, 031)	0.957	0.961	0.050	0.923
Model D	309.72 (542)	0.000 ^1^	1.022	1.000	0.036	0.936

Note. Model A: Unidimensional model. Model B: Correlated factor model. Model C: Higher-order factor model. Model D: Bifactor model. ^1^ Cannot compute boundary of confidence interval.

**Table 2:** Model fit indices for invariance testing of the Covitality Model. Sample 1.

	S-Bχ^2^	*Df*	SRMR	RMSEA [CI]	CFI	DCFI	Mc	DMc	GHI	DGHI
M1.	293.21	98	0.053	0.062 [0.054, 0.070]	0.935	—	0.911	—	0.94	—
M2.	321.32	106	0.065	0.063 [0.055, 0.070]	0.928	−0.007	0.902	−0.009	0.93	−0.01
M3.	328.29	110	0.075	0.062 [0.054, 0.069]	0.927	−0.008	0.901	−0.01	0.93	−0.01
M4.	298.66	112	0.078	0.044 [0.036, 0.053]	0.965	0.03	0.948	0.037	0.97	0.03
M5.	409.92	117	0.078	0.055 [0.047, 0.063]	0.947	0.012	0.921	0.01	0.94	0.00

Note. S-Bχ^2^ = Satorra-Bentler scaled chi square statistic. SRMR = standardized root-mean-square residual. RMSEA = robust root-mean-square error of approximation. CI = confidence interval. DCFI = difference in robust comparative fit indices between baseline model. Mc: McDonald’s Noncentrality Index. DMc: differences in Mc between baseline model. GH: Gamma Hat Index. DGH: differences in GHI between baseline model. M1 = Model 1 (baseline model) configure invariance. M2 = Model 2: M1+metric invariance first order factors. M3 = Model 3: M2+ metric invariance second order factor. M4: M3+ scalar invariance (observed variable intercepts). M5: M4+ full scalar invariance (latent factors intercepts).

**Table 3:** Bivariate correlation analyses between SESH-S subscales (Sample 2).

	1	2	3	4	5	6	7	8	9	10	11	12	A	B	C	D
1	1.00															
2	0.56 **	1.00														
3	0.32 **	0.31 **	1.00													
4	0.35 **	0.31 **	0.29 **	1.00												
5	0.20 **	0.32 **	0.16 *	0.16 *	1.00											
6	0.23 **	0.27 **	0.11	0.14 *	0.14 *	1.00										
7	0.34 **	0.30 **	0.15 *	0.18 **	0.14 *	0.18 **	1.00									
8	0.15 *	0.19 **	0.19 **	0.23 **	0.22 **	0.21 **	0.40 **	1.00								
9	0.21 **	0.25 **	0.15 *	0.18 **	0.11	0.15 *	0.41 **	0.30 **	1.00							
10	0.45 **	0.44 **	0.12	0.33 **	0.32 **	0.27 **	0.18 **	0.27 **	0.18 **	1.00						
11	0.40 **	0.42 **	0.17 *	0.30 **	0.36 **	0.32 **	0.18 **	0.24 **	0.19 **	0.56 **	1.00					
12	0.43 **	0.41 **	0.18 **	0.26 **	0.36 **	0.24 **	0.23 **	0.27 **	0.21 **	0.53 **	0.52 **	1.00				
A	0.77 **	0.78 **	0.76 **	0.41 **	0.29 **	0.25 **	0.33 **	0.23 **	0.26 **	0.41 **	0.41 **	0.42 **	1.00			
B	0.39 **	0.46 **	0.28 **	0.64 **	0.71 **	0.61 **	0.25 **	0.33 **	0.22 **	0.47 **	0.50 **	0.44 **	0.48 **	1.00		
C	0.30 **	0.32 **	0.22 **	0.26 **	0.21 **	0.24 **	0.76 **	0.77 **	0.75 **	0.28 **	0.27 **	0.32 **	0.36 **	0.36 **	1.00	
D	0.51 **	0.51 **	0.19 **	0.36 **	0.41 **	0.34 **	0.24 **	0.31 **	0.23 **	0.82 **	0.85 **	0.82 **	0.50 **	0.56 **	0.35 **	1.00
X	0.65 **	0.69 **	0.47 **	0.54 **	0.53 **	0.46 **	0.50 **	0.52 **	0.46 **	0.69 **	0.71 **	0.69 **	0.77 **	0.77 **	0.65 **	0.83 **

Note. 1 = Self-Efficacy, 2 = Self-Awareness, 3 = Persistence, 4 = School support, 5 = Family coherence, 6 = Peer support, 7 = Emotional regulation, 8 = Empathy, 9 = Behavioral self-control, 10 = Optimism, 11 = Zest, 12 = Gratitude, A = Belief in self, B = Belief in other, C = Emotional competence, D = Engaged living, X = Covitality. * *p* < 0.05. ** *p* < 0.01. *** *p* < 0.001.

**Table 4:** Bivariate correlation analyses between dependent and independent variables (Sample 2).

Well-Being and Distress Measures	Covitality	Belief in Self	Belief in Others	Emotional Competence	Engaged Living
MHC-SF total score	0.70 **	0.64 **	0.55 **	0.30 **	0.60 **
Hedonic well-being	0.52 **	0.47 **	0.42 **	0.15 *	0.50 **
Eudemonic well-being	0.70 **	0.65 **	0.55 **	0.32 **	0.58 **
Psycho	0.68 **	0.63 **	0.54 **	0.33 **	0.55 **
Social	0.60 **	0.56 **	0.48 **	0.26 **	0.51 **
KIDSCREEN-10 total score	0.63 **	0.55 **	0.48 **	0.22 **	0.60 **
SDQ total score	−0.41 **	−0.40 **	−0.33 **	−0.26 **	−0.26 **
Prosocial behavior	0.46 **	0.30 **	0.41 **	0.47 **	0.27 **
Externalizing symptoms	−0.30 **	−0.28 **	−0.24 **	−0.34 **	−0.09
Internalizing symptoms	−0.36 **	−0.35 **	−0.29 **	−0.11	−0.32 **

Note. MHC-SF = Mental Health Continuum-Short Form. SDQ = Strengths and Difficulties Questionnaire. * *p* < 0.05. ** *p* < 0.01. *p* < 0.001.

## Supplementary Materials

The following are available online at https://www.mdpi.com/1660-4601/16/24/4982/s1. Document S1: Social Emotional Health Survey-Secondary (SEHS-S). Spanish Language Version. Document S2: SEHS-S correction. Document S3: Description of Covitality indicators.
