# Supplementary material for: Social Emotional Health Survey-Secondary (SEHS-S): A Universal Screening Measure of Social-Emotional Strengths for Spanish-Speaking Adolescents
Source: Int J Environ Res Public Health. 2019 Dec 7;16(24):4982. doi: 10.3390/ijerph16244982 (PMC6950097; doi:10.3390/ijerph16244982)
Supplement: Supplementary file 1 [file ijerph-16-04982-s001.pdf]

Supplementary materials:

Document S1. Social Emotional Health Survey-Secondary, Spanish Language Version

Marca la respuesta que consideres verdadera con un círculo. Por favor seleccione la respuesta apropiada para cada concepto.

1= Para nada cierto // 2=Un poco cierto // 3=Más o menos cierto // 4=Muy cierto.

1. Puedo solucionar mis problemas.	1	2	3	4
2. Puedo hacer casi todo si lo intento.	1	2	3	4
3. Hay muchas cosas que hago bien.	1	2	3	4
4. Mi vida tiene un propósito.	1	2	3	4
5. Entiendo mis estados de ánimo y sentimientos.	1	2	3	4
6. Entiendo por qué hago lo que hago.	1	2	3	4
7. Cuando no entiendo algo, le pregunto al profesor/a una y otra vez hasta entenderlo.	1	2	3	4
8. Intento responder todas las preguntas que se hacen en clase	1	2	3	4
9. Cuando intento resolver un problema de matemáticas, no me detengo hasta encontrar una solución.	1	2	3	4
10. En mi escuela hay un/a profesor/a o algún otro adulto/a que quiere que lo haga lo mejor que pueda.	1	2	3	4
11. En mi escuela hay un/a profesor/a o algún otro adulto/a que me escucha cuando tengo algo que decir.	1	2	3	4
12. En mi escuela hay un/a profesor/a o algún otro adulto/a que cree que tendré éxito.	1	2	3	4
13. Los miembros de mi familia realmente se ayudan y se apoyan unos/as a otros/as.	1	2	3	4
14. Hay una sensación de unidad en mi familia.	1	2	3	4
15. Mis familiares se llevan bien entre ellos/as.	1	2	3	4
16. Tengo un/a amigo/a de mi edad que realmente se preocupa por mí.	1	2	3	4
17. Tengo un/a amigo/a de mi edad que habla conmigo sobre mis problemas.	1	2	3	4
18. Tengo un/a amigo/a de mi edad que me ayuda cuando estoy pasando un mal rato.	1	2	3	4
19. Me hago responsable de mis acciones.	1	2	3	4
20. Admito cuando cometo algún error.	1	2	3	4
21. Puedo tolerar cuando me dicen que “no”.	1	2	3	4
22. Me siento mal cuando le hieren los sentimientos a alguien.	1	2	3	4
23. Trato de entender por lo que pasan otras personas.	1	2	3	4
24. Trato de entender cómo piensan y sienten otras personas.	1	2	3	4
25. Puedo esperar por lo que quiero.	1	2	3	4
26. No molesto a los demás cuando están ocupados.	1	2	3	4
27. Pienso antes de actuar.	1	2	3	4
28. Espero divertirme mucho cada día.	1	2	3	4
29. Usualmente espero tener un buen día.	1	2	3	4
30. En general espero que me pasen más cosas buenas que malas.	1	2	3	4

1=Para nada // 2=Muy poco // 3=Algo // 4=Mucho //5=Extremadamente.

31. ¿Cómo de lleno/a de energía te sientes ahora?	1	2	3	4	5
32. ¿Cómo de activo/a te sientes ahora?	1	2	3	4	5
33. ¿Cómo de animado/a te sientes ahora?	1	2	3	4	5
34. ¿Cómo de satisfecho/a te has sentido desde el día de ayer?	1	2	3	4	5
35. ¿Cómo de agradecido/a te has sentido desde el día de ayer?	1	2	3	4	5
36. ¿Cómo de apreciativo/a (que aprecia o valora lo que le rodea) te has sentido desde el día de ayer?	1	2	3	4	5

© Spanish version by Piqueras, Rodríguez-Jiménez, Marzo, Rivera-Riquelme, Martínez-González, Falcó, & Furlong

Document S2. Correction syntax for the SEHS-S

12 FIRST-ORDER SUBSCALES:

**Self-Efficacy**	SEHS-S1 + SEHS-S2 + SEHS-S3.
**Self-Awareness**	SEHS-S4 + SEHS-S5 + SEHS-S6.
**Persistence**	SEHS-S7 + SEHS-S8 + SEHS-S9.
**Teacher Support**	SEHS-S10 + SEHS-S11 + SEHS-S12.
**Family Coherence**	SEHS-S13 + SEHS-S14 + SEHS-S15.
**Peer Support**	SEHS-S16 + SEHS-S17 + SEHS-S18.
**Emotional Regulation**	SEHS-S19 + SEHS-S21 + SEHS-S21.
**Empathy**	SEHS-S22 + SEHS-S23 + SEHS-S24.
**Self-Control**	SEHS-S25 + SEHS-S26 + SEHS-S27.
**Optimism**	SEHS-S28 + SEHS-S29 + SEHS-S30.
**Zest**	SEHS-S31 + SEHS-S32 + SEHS-S33.
**Gratitude**	SEHS-S34 + SEHS-S35 + SEHS-S36.

FOUR SECOND-ORDER FACTORS:

**Belief in self**	Self-Efficacy + Self-Awareness + Persistence.
**Belief in others**	Teacher Support + Family Coherence + Peer Support.
**Emotional Competence**	Emotional Regulation + Empathy + Self-Control.
**Engaged living**	Optimism + Zest + Gratitude.

THIRD-ORDER GENERAL FACTOR: COVITALITY:

**Covitality**	Belief-in-self + Belief-in-other + Emotional Competence + Engaged Living.

Document S3. Description of Covitality indicators

Covitality Model

Covitality Indicator	Definition
**BELIEF IN SELF**	
**Self-Efficacy**	Belief in one's ability to succeed in specific situations or when performing a task or is confidence in one's ability to achieve intended results [1].
**Self-Awareness**	Recognition of one's own emotions and values, and their impact on behavior, as well as accurate self-assessment of strengths and challenges [2].
**Persistence**	Working with dedication to achieve one's own objectives, maintaining interest in the face of difficulty [3].
**BELIEF IN OTHERS**	
**Teacher Support**	Assess the careful and helpful nature of one's relationships with teachers [4].
**Family Coherence**	Feeling that the family is united and supportive [4].
**Peer Support**	Assess the careful and helpful nature of one's relationships with [4].
**EMOTIONAL COMPETENCE**	
**Emotional Regulation**	The ability to manage our emotions and our efforts to control what emotions we feel, when we feel them, and how we experience and express them [5].
**Empathy**	Ability to share another person's feelings or motivations and understand their point of view [6].
**Self-Control**	Ability to control one's own behaviors or thoughts in difficult situations in order to obtain a reward or avoid punishment [7].
**ENGAGED LIVING**	
**Optimism**	Tendency to believe that good things will come and to have a positive vision of the future [8].
**Zest**	Approach life with enthusiasm and energy [9].
**Gratitude**	Sensing thankfulness that arises in response to receiving any kind of personal benefit as a result of any transactional means [10].

References

1. Bandura, A.; Barbaranelli, C.; Capara, G.V.; Pastorelli, C. Multifaceted impact of self-efficacy beliefs on academic functioning. *Child Dev.* **1996**, *67*, 1206–1222. doi:10.2307/1131888.
2. Abrams, D.; Brown, R. Self-Consciousness and social identity: Self-regulation as a group member. *Soc. Psychol. Q.* **1989**, *52*, 311–318. doi:10.2307/2786994.
3. Duckworth, A.L.; Peterson, C.; Matthews, M.D.; Kelly, D.R. Grit: Perseverance and passion for long-term goals. *Pers. Process. Individ. Differ.* **2007**, *92*, 1087–1101. doi:10.1037/0022-3514.92.6.1087.
4. Farmer, T.W.; Farmer, E. Social relationships of students with exceptionalities in mainstream classrooms: Social networks and homophily. *Except. Child.* **1996**, *62*, 431–450. doi:10.1177/001440299606200504.
5. Fry, M.D.; Guivernau, M.; Kim, M.S.; Newton, M.; Gano-Overway, L.A.; Magyar, M. Youth perceptions of a caring climate, emotional regulation, and psychological well-being. *Sport Exerc. Perform. Psychol.* **2012**, *1*, 44–57. doi:10.1037/a0025454.
6. Garaigordobil, M. Effects of a psychological intervention on factors of emotional development during adolescence. *Eur. J. Psychol. Assess.* **2004**, *20*, 66–80. doi:10.1027/1015-5759.20.1.66.
7. Hofer, J.; Busch, H.; Kartner, J. Self-regulation and well-being: The influence of identity and motives. *Eur. J. Personal.* **2011**, *25*, 211–224. doi:10.1002/per.789.
8. Utsey, S.O.; Hook, J.N.; Fischer, N.; Belvet, B. Cultural orientation, ego resilience, and optimism as predictors of subjective well-being in African Americans. *J. Posit. Psychol.* **2008**, *3*, 202–210. doi:10.1080/17439760801999610.
9. Park, N.; Peterson, C. Moral competence and character strengths among adolescents: The development and validation of the Values in Action Inventory of Strengths for Youth. *J. Adolesc.* **2006**, *29*, 891–909. doi:10.1016/j.adolescence.2006.04.011.
10. Emmons, R.A. *Thanks!*; Houghton Mifflin: New York, NY, USA, 2007.
